# The Identification Distinct Antiviral Factors Regulated Influenza Pandemic H1N1 Infection

**DOI:** 10.1155/2024/6631882

**Published:** 2024-01-09

**Authors:** Baoxin Wang, Hao Zheng, Xia Dong, Wenhua Zhang, Junjing Wu, Hongbo Chen, Jing Zhang, Ao Zhou

**Affiliations:** ^1^School of Animal Science and Nutritional Engineering, Laboratory of Genetic Breeding, Reproduction and Precision Livestock Farming, Wuhan Polytechnic University, Wuhan 430023, Hubei, China; ^2^Hubei Provincial Center of Technology Innovation for Domestic Animal Breeding, Wuhan 430023, Hubei, China; ^3^Hubei Key Laboratory of Animal Embryo and Molecular Breeding, Institute of Animal Husbandry and Veterinary, Hubei Provincial Academy of Agricultural Sciences, Wuhan, China

## Abstract

Influenza pandemic with H1N1 (H1N1pdms) causes severe lung damage and “cytokine storm,” leading to higher mortality and global health emergencies in humans and animals. Explaining host antiviral molecular mechanisms in response to H1N1pdms is important for the development of novel therapies. In this study, we organised and analysed multimicroarray data for mouse lungs infected with different H1N1pdm and nonpandemic H1N1 strains. We found that H1N1pdms infection resulted in a large proportion of differentially expressed genes (DEGs) in the infected lungs compared with normal lungs, and the number of DEGs increased markedly with the time of infection. In addition, we found that different H1N1pdm strains induced similarly innate immune responses and the identified DEGs during H1N1pdms infection were functionally concentrated in defence response to virus, cytokine-mediated signalling pathway, regulation of innate immune response, and response to interferon. Moreover, comparing with nonpandemic H1N1, we identified ten distinct DEGs (AREG, CXCL13, GATM, GPR171, IFI35, IFI47, IFIT3, ORM1, RETNLA, and UBD), which were enriched in immune response and cell surface receptor signalling pathway as well as interacted with immune response-related dysregulated genes during H1N1pdms. Our discoveries will provide comprehensive insights into host responding to pandemic with influenza H1N1 and find broad-spectrum effective treatment.

## 1. Introduction

With the evolution, reassortment, and transmission of influenza virus, influenza pandemics caused severe pneumonia and higher mortality, leading to public health emergence and economic losses [1]. Influenza H1N1 strains have resulted in two pandemics in history spreading worldwide and killing many individuals [2, 3]. H1N1pdm1918 and H1N1pdm2009 originated from a series of reassortments among avian, swine, and human influenza viruses and then transmitted to humans, leading to acute lung injury [4, 5]. Influenza virus reassortments usually affect the efficacy of vaccination, which is the most efficient approach to prevent and control influenza circulation [6], and only four anti-influenza drugs (oseltamivir, zanamivir, peramivir, and baloxavir) were used, although the usage of these drugs may lead to the emergence of resistant influenza strains [7–11]. Therefore, novel broad-spectrum treatments are needed to be explored and developed.

A large number of host factors and cellular processes, for example, host dependency factors, host restriction factors, apoptosis, and autophagy are involved in the replication cycle of influenza virus [12–16]. It was all known that faced with infection, host will quickly respond to virus clearance and tissue function maintenance for the host survives by releasing antiviral signalling [17]^.^ Host antiviral responses activated by influenza virus infection, in turn, can prevent viral infection by inhibiting the fusion of viral and host membranes, inducing viral protein degradation and strengthening the innate immune response and antiviral signalling of MAVS [18–20]. Moreover, host apoptosis directly targets influenza virus-infected cells [21] and autophagy induces damaged oranges containing viral particles into the lysosome for viral elimination [22]. But the virus can employ host compounds and processes to promote its replication and induce lung injury. Sialic acid receptor on the host cell surface is a key for the initiation of influenza virus infection that depends on cellular endocytosis [23, 24]. Host factors CMAS and ST3GAL4 knockout inhibited the synthesis of sialic acid receptors [25]. Glucosylceramidase (GBA) regulates influenza virus entry and cellular endocytosis [26]. Additionally, influenza virus infection induces the release of proinflammatory cytokines that promote influenza virus-related lung injury [27]. These data indicate that the interaction between the host and virus is complex. Thus, the releasing mechanisms of virus-host interaction are particularly important, and analyses of the lung transcriptomic pattern in response to viral infections are a useful paradigm.

In this study, we performed an integrative analysis of transcriptomic expression profile data of mouse lung to assess host response patterns to different H1N1pdm strains and identify differentially expressed genes (DEGs). Then, gene ontology and pathway enrichment analyses were performed to clarify the function of these assessed DEGs, and protein-protein interaction (PPI) network analysis was also conducted and revealed key genes. We identified distinct dysregulated genes during H1N1pdms infection. These results contribute to understanding the host response mechanisms to H1N1pdm virus.

## 2. Methods

### 2.1. Data Collection and Identification of DEGs

Microarray datasets, including GSE43764, GSE40091, GSE63786, GSE67241, GSE70882, GSE38112, GSE70445, GSE44595, GSE70502, GSE57008, GSE99189, GSE99190, GSE77600, GSE158270, and GSE62169 [28–39] (Table 1), were selected based on infection time and strains. Moreover, these datasets contained at least three duplicate samples in each group. The raw expression matrix files were downloaded by using GEOquery package in R, and then the mRNA expression profile datasets of lung infected with influenza H1N1 virus were analysed with the limma package [40] to identify differentially expressed genes (DEGs) based on the selection criteria of adj. *p* value <0.05 and the absolute value of log2 (fold change) >1. A heatmap of the DEGs in R software was drawn with the pheatmap package, and the Venn diagram for the overlapped DEGs was drawn with the VennDiagram package.

### 2.2. Analysis of Gene Ontology and Pathway Enrichment

To explain the role of DEGs in response to H1N1 virus infection, gene ontology (GO) enrichment analysis for the biological process was performed using the cluster profiler package [41] in R software, and a *p* value <0.05 was considered statistically significant. Reactome pathway and Kyoto Encyclopedia of Genes and Genomes (KEGG) pathway enrichment analysis were combined to evaluate the functional pathways of the H1N1-associated genes by KEGG and Reactome databases with *p* value <0.05 [42, 43].

### 2.3. Protein-Protein Interaction (PPI) Analysis and Network Construction

The common DEGs were selected for protein-protein interaction analysis by using the STRING database (https://string-db.org/). Then, the cluster analysis of the PPI network was performed with the MCL inflation parameter in STRING. The significant cluster networks were exported to Cytoscape for further visualization processing [44].

## 3. Results

### 3.1. Transcriptomic Profiles of the H1N1pdm2009-Infected Mouse Lung

**Figure 1:**
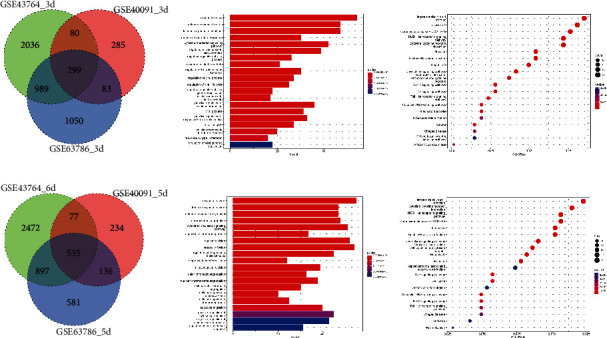
Identification and function enrichment analysis of differentially expressed genes (DEGs) of the lung during H1N1pdm2009 infection. (a, d) Gene expression profile analyses of the infected lung with A/California/04/2009 strains indicating the common and distinct gene sets in 3 day-postinfection and 5/6 day-postinfection via Venn diagram; (b, e) Gene ontology analysis showing the biological process of DEGs; (c, f) pathway enrichment analysis of DEGs.

**Figure 2:**
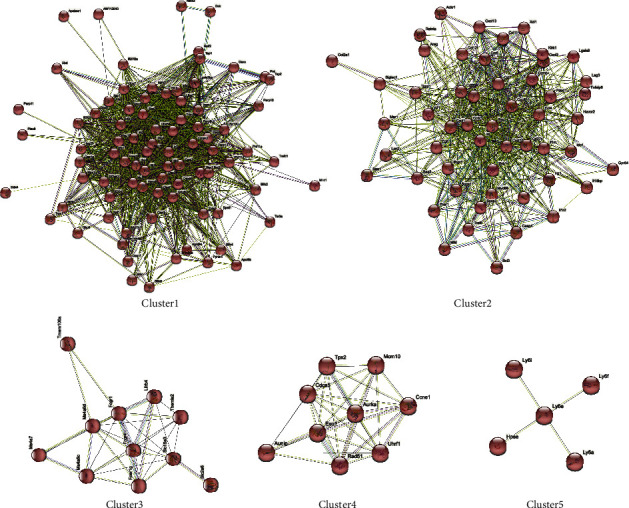
The protein-protein interaction (PPI) clusters of upregulated DEGs based on the MCL inflation parameter in the STRING database. 173 of the upregulated DEGs were divided into five significant clusters.

The mRNA expression profiles of the mouse lung infected with a pandemic influenza virus (H1N1pdm09) were performed in here. Three independent datasets (GSE43764, GSE40091, and GSE63786) with A/California/04/2009 H1N1 infection (a subtype of H1N1pdm09) were integrated to identify differentially expressed genes (DEGs) using the limma package. The result showed that a subtype of H1N1pdm09, A/California/04/2009 H1N1, produced numerous DEGs at three-day postinfection (3 dpi) and a time-dependent increase of DEGs was detected after 5/6 dpi (Figures 1(a) and 1(d), Supplementary data1). Moreover, to look for the functional characteristics of these DEGs, gene ontology (GO) classification and pathway enrichment analysis were performed. Consistently, we found that H1N1pdm09-induced DEGs in both 3 dpi and 5 dpi were mainly associated with the response to virus and innate immune process, where a part of DEGs showed a role in organelle fission and nuclear division process in 5/6 dpi (Figures 1(b) and 1(e)). KEGG pathway analysis indicated that these DEGs were overrepresented in pathways associated with viral protein interaction with cytokine and cytokine receptors, NOD-like receptor signalling, and influenza A (Figures 1(c) and 1(f)). Furthermore, collecting all DEGs in 3 dpi and 5/6 dpi, we identified 231 common DEGs, including 19 downregulated and 209 upregulated genes in response to H1N1pdm09 infection (Supplementary data1). The downregulation was observed for genes (1500035N22Rik, 1700012B09Rik, 2410066E13Rik, Aass, Abcg5, Asgr1, Cd207, Ces1f, Cyp2a4, Cyp4f15, F2, Fabp1, Fmo3, Hepacam2, Hmgcs2, PiPOx, Pon1, Scgb1c1, and Uox) involved in the L-lysine catabolic process to acetyl-CoA, acetyl-CoA metabolic, carboxylic acid catabolic, and small molecule catabolic process, while the biological process terms of the upregulated genes were enriched in response to virus, negative regulation of the viral process, and regulation of innate immune response. With MCL inflation parameters based on the STRING database, protein-protein interaction (PPI) networks were generated (*p* value <1.0*e* − 16). 173 of the upregulated DEGs were divided into five significant clusters (Figure 2). STAT1, Cxcl10, and IRF7 nodes have the highest degree of connectivity (degree ≥ 90) in the PPI networks.

**Figure 3:**
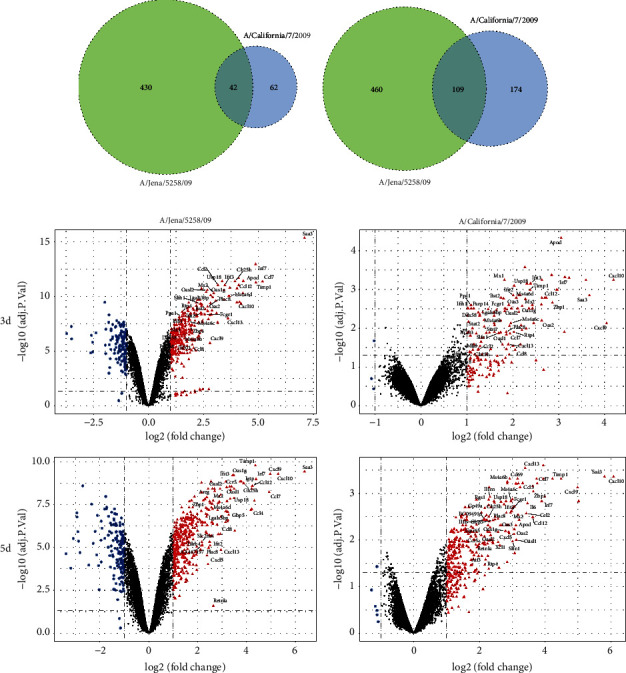
Gene expression profile analyses of the infected lung with other H1N1pdm2009 strains. (a, b) Gene expression profile analyses of the infected lung with three other H1N1pdm2009 strains indicating the common and distinct gene sets in 3 dpi and 5 dpi via Venn diagram; (c) volcano plot representation of DEGs in three difference microarray datasets (GSE63786, GSE67241, and GSE70882). Red and blue colours indicate the genes increased or decreased expression, respectively. The overlapped DEGs are separately displayed.

**Figure 4:**
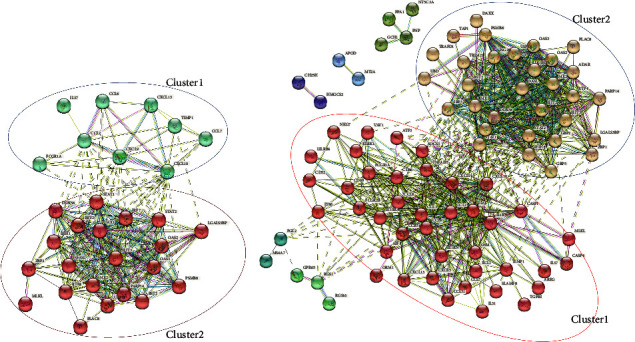
Functional enrichment and PPI network of the overlapped DEGs. (a) 15 DEGs in 3 dpi were constructed using the STRING database and divided into one cluster and (b) PPI network of 69 DEGs in 5 dpi was constructed, and the roles of these genes were enriched in defense response.

In addition, to identify whether other subtypes of H1N1pdm09 strain show a similar transcriptomic profile, three independent microarray datasets of the mouse lung responding to two different strains of H1N1pdm09 (A/Jena/5258/09 and A/California/7/2009) infection were jointly analysed (Supplementary data2). A similar conclusion was reached that the number of overlapped DEGs (42 in 3 dpi and 109 in 5 dpi) in all two datasets was correlated with time points after infection (Figures 3(a) and 3(b), Supplementary data2). We found that all overlapped DEGs were upregulated and displayed in a volcano plot (Figure 3(c)). Furthermore, functional enrichment and PPI network analysis of these DEGs indicated that genes in Cluster1 were involved in the cytokine-mediated signalling pathway, neutrophil chemotaxis, and inflammatory response, when genes in Cluster2 mainly played a role in the type I interferon signalling pathway, defence response, and innate immune response in both 3 dpi and 5 dpi (Figures 4(a) and 4(b)). Integrated with gene expression profiles in different strains of H1N1pdm2009, we finally identified 38 and 87 overlapped DEGs in all analysed datasets infected with H1N1pdm09 strains at 3 dpi and 5 dpi, respectively (Supplementary data3).

### 3.2. Differential Gene Expression Profile between the H1N1pdm1918-Infected Lung and Healthy Controls

**Figure 5:**
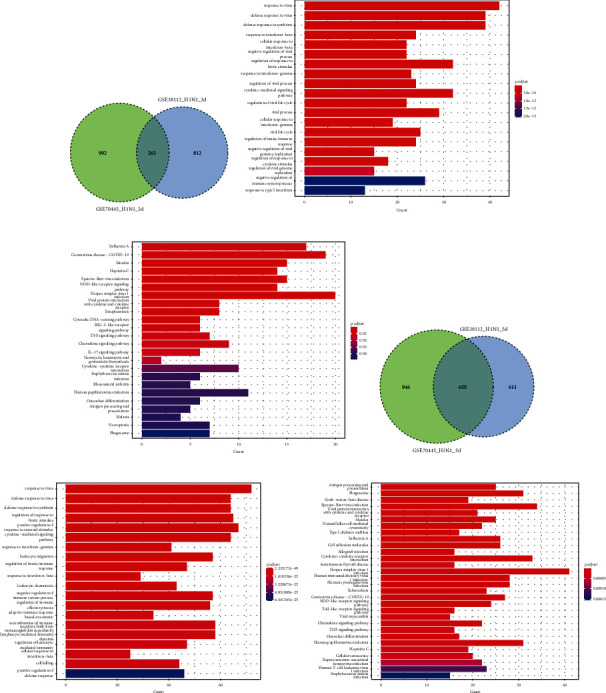
Global transcriptomic profiles change of the lung during H1N1pdm1918 infection. (a, d) Heatmap of differentially expressed patterns of genes in the H1N1pdm1918-infected lung from two microarray datasets (GSE38112 and GSE70445); (b, e) gene ontology analysis showing the biological process of DEGs; (c, f) KEGG pathways of the DEGs. Significant top 20 enriched by differentially expressed genes were shown.

Another pandemic influenza virus 1918 H1N1 strain (H1N1pdm1918) caused the deadly influenza pandemic and severe lung injury. To explore the regulatory mechanism of lung in-host defence against H1N1pdm1918 virus infection, we performed a comparative gene expression profiling by using the publicly available array expression profiling datasets (GSE38112 and GSE70445). A direct comparison analysis of up or down trends in expression showed that there were 263 and 650 overlapped DEGs in 3 dpi and 5 dpi, respectively (Figures 5(a) and 5(d), Supplementary data4).

**Figure 6:**
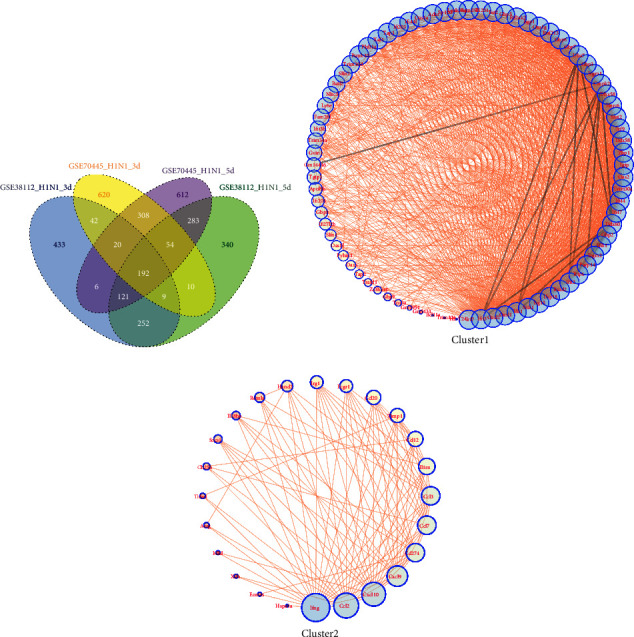
The overlapped DEGs in H1N1pdm1918. (a) Venn diagram of the overlapped genes between different data. A total of 192 genes were common to the H1N1pdm1918-infected lung at 3 dpi and 5 dpi; (b, c) the common DEGs were divided into two clusters and visualized using Cytoscape.

Importantly, H1N1pdm1918-induced DEGs represented the similar function characteristic with those DEGs in H1N1pdm09 (Figures 5(b) and 5(e)). Compared with DEGs in 3 dpi that were mostly involved in the innate immune process, those DEGs in 5 dpi were enriched in adaptive immunity-related antigen processing and presentation and phagosome process (Figures 5(c) and 5(f)). To reveal whether there are common dysregulated genes during H1N1pdm1918 virus infection, we continuously analysed time-course gene expression profiling in 3 dpi and 5 dpi. 192 overlapped DEGs were identified with Venn diagrams (Figure 6(a) and Supplementary data4) and significantly enriched in host defence, immune system, and interferon signalling. Additionally, the differential protein-protein interaction network was constructed and showed two significant clusters: one cluster (80 genes) enriched interferon signalling and antiviral mechanism by IFN-stimulated genes, another cluster (24 genes) enriched chemokine receptor binding chemokines and regulating of IFNG signalling (Figures 6(b) and 6(c)). By integrating all DEGs in H1N1pdms, we acquired 32 and 74 dysregulated genes at 3 dpi and 5 dpi, respectively (Supplementary data4).

### 3.3. Identification of Distinct DEGs in H1N1pdm Strains Compared with Nonpandemic H1N1

**Figure 7:**
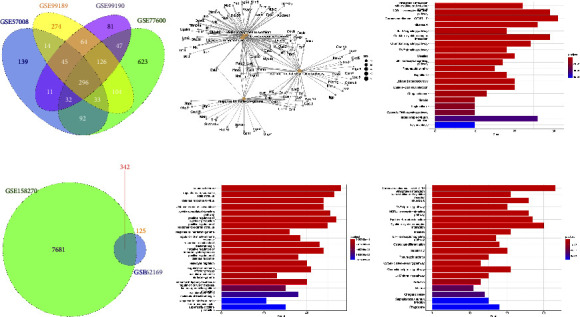
Identification and function enrichment analysis of differentially expressed genes (DEGs) of the lung during A/PR/8/34 infection. (a, d) Gene expression profile analyses of the infected lung with A/PR/8/34 strains indicating the common and distinct gene sets in 3 dpi and 5 dpi via Venn diagram; (b, e) gene ontology analysis showed the biological process of DEGs; (c, f) cell signalling pathway analyses showing pathway enrichment of the DEGs. Significant top 20 enriched by differentially expressed genes were shown.

**Figure 8:**
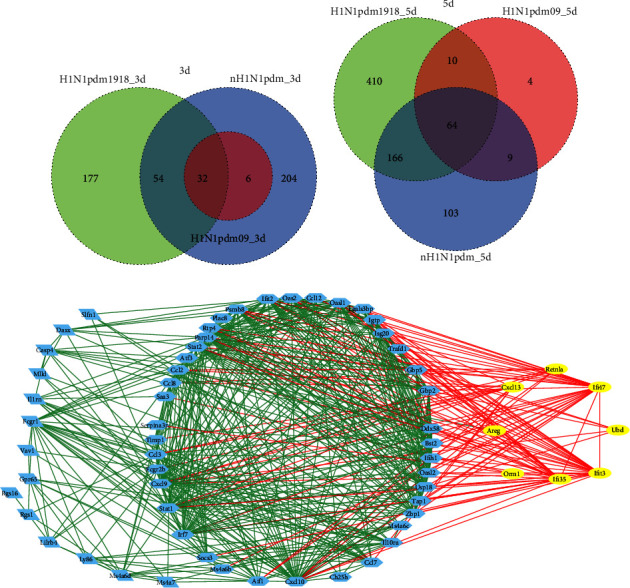
Identification of the distinct DEGs in H1N1pdms strains compared with nH1N1pdms. (a, b) Venn diagram showing the distinct DEGs in H1N1pdms at 3 dpi and 5 dpi, respectively. (c) The distinct DEGs in H1N1pdms interacted with H1N1pdms-induced dysregulation genes related with immune response. Yellow circle represents the identified distinct DEGs; red line represents the interaction with distinct DEGs.

To make clear H1N1pdms-induced distinct host responses, gene expression profiles of lungs infected with nonpandemic H1N1 (nH1N1pdm) were analysed and then compared with the DEGs induced by H1N1pdms. Similarly, a direct comparison of up or down trends showed that infection of nH1N1pdm triggered a strong and persistent innate immune response due to the production of many innate immune-related upregulated genes (Figures 7(a) and 7(d), Supplementary data5). The biological process enrichment analysis of 296 DGEs in 3 dpi and 342 DEGs in 5 dpi showed significant enrichment of response to virus, defence response to virus, and response to interferon-gamma (Figures 7(b) and 7(e)). KEGG analysis showed a significant enrichment of upregulated genes involved in the NOD-like receptor signalling pathway, Toll-like receptor signalling pathway, TNF signalling pathway, and cytokine-cytokine receptor interaction (Figures 7(c) and 7(f)). Besides, we found that DEGs in H1N1pdms infection were completely present in nonpandemic H1N1 infection at 3 dpi, while only ten distinct DEGs (AREG, CXCL13, GATM, GPR171, IFI35, IFI47, IFIT3, ORM1, RETNLA, and UBD) in H1N1pdm strains infection were identified in comparison with nonpandemic H1N1 at 5 dpi (Figures 8(a) and 8(b)), suggesting that H1N1pdm induces host distinct response in the later stage of infection. Moreover, we found that these distinct DEGs were continuously upregulated in H1N1pdms infection, while there was no change or rapid up- and -downregulation in nH1N1pdms infection. Furthermore, the biological process of these distinct DEGs was involved in immune response and cell surface receptor signalling pathway, and these distinct DEGs can interact with immune response-related dysregulated genes (Figure 8(c)).

## 4. Discussion

H1N1 influenza pandemic (H1N1pdms) causes severe public health emergency, resulting in severe pneumonia and high mortality rates. The different strains of H1N1pdms can show distinct infection patterns and interaction with the host, suggesting that the study of H1N1pdms-host interaction is essential. Due to higher genetic mutations and reassortment, the different strains of H1N1pdms can utilize different mechanisms to induce host injury. Thus, explaining the host response to H1N1pdms infection and identifying critical genes and signalling pathways will provide novel treatment strategies in influenza pandemic. In this study, we performed multiple gene expression profiles and used bioinformatical approaches to investigate host responses to different H1N1pdms and identify distinct differentially expressed genes in H1N1pdms infection compared with nonpandemic H1N1.

H1N1pdms elicit acute hyperinflammatory response, causing lung damage and respiratory failure as well as death [45], and host resistance and tolerance to H1N1pdms-induced lung injury refer to host genes' expression level [46]. However, the mechanisms of hyperinflammatory activation during H1N1pdms infection and the interaction of host-H1N1pdms are unclear. Our transcriptomic profiling and biological processes analysis explored that H1N1pdms-induced dysregulated genes were mainly involved in defence to infection, chemokine receptors binding chemokines, and regulating of IFNG signalling. Importantly, most of ten identified distinct DEGs (AREG, CXCL13, GATM, GPR171, IFI35, IFI47, IFIT3, ORM1, RETNLA, and UBD) in H1N1pdms infection in our study are involved in host response to viruses, and the discovery of these molecular biomarkers may provide new insights into diagnosis and treatment against H1N1pdms infection. IFI35, IFI47, and IFIT3 are associated with the immune and defence process. IFI35 can increase H5N1 influenza disease and has been identified as a promising biomarker and therapeutic target for syndromes induced by SARS-CoV-2 or influenza virus [47, 48].

Amphiregulin (AREG) is an epidermal growth factor that plays an important role in regulating virus-infected lung repair [49]. AREG expression has been reported in epithelial cell layers and various immune cells, including dendritic cells, neutrophiles, and CD4^+^ T cells [50, 51], and is constitutively upregulated in response to inflammation or infection [52]. AREG can promote alveolar remodelling and integrity during influenza virus infection. Innate lymphoid cells (ILCs) that are critical in immune response and tissue homeostasis can produce AREG, which in turn restores lung function and airway remodel [53]. Previous studies have shown that influenza viruses bind to sialic acid receptors and then lead to the activation of EGFR, promoting virus entry [54], suggesting that AREG-EGFR signalling could function in host immune response to influenza virus and tissue tolerance. In addition, C-X-C motif chemokine ligand 13 (CXCL13) is also involved in receptor-mediated signalling pathways, except for its proinflammatory function [55]. HIV-1-infected and COVID-19 patients have higher levels of plasma and serum CXCL13 concentration, and CXCL13 has been identified as a biological signature of COVID-19 patients and HIV-1 patients [56–58]. Moreover, high levels of CXCL13 expression have been proved to be associated with pulmonary fibrosis that is the prominent feature of infection with 2009 pandemic influenza A (H1N1) virus [59, 60], suggesting that CXCL13 may play an important role in pulmonary diseases caused by influenza virus infection and still need to be further investigated. Resistin-like alpha (RETNLA), a cysteine-rich secreted family of Fizz/Resistin-like molecules and a M2 macrophage marker that modulates lung fibrosis and inflammation, has been revealed to act as a marker of activated macrophages and involved in the immune response-induced pulmonary vascular remodelling [61–63]. It is all known that the mRNA levels of RETNLA can reflect M2 macrophage polarization and influenza virus infection-induced cell apoptosis [64]. Our result shows the upregulated RETNLA expression in H1N1pdms, indicating that H1N1pdms infection may increase M2 macrophage apoptosis. In addition, overexpression of RETNLA can decrease allergic lung inflammation by reducing infiltration of immune cells and Th2 cytokine production, suggesting that the host may increase RETNLA expression to trigger M2 macrophage polarization and promote lung repair during H1N1pdms infection [65]. A previous study has shown that glycine amidinotransferase (GATM) was upregulated in M2-polarized macrophages. GATM deletion inhibited the expression of RETNAL and blocked M2 polarization [66]. Based on these, we speculate that GATM may regulate RETNLA to affect M2 macrophage polarization during H1N1pdms infection.

The interaction between DEGs and transcription factors (TFs) was explored to know about how the DEGs regulate influenza virus at the transcriptional level. Our analysis of the TFs-DEGs network found that BATF2 was the most significant TF as the regulator of DEGs. We found that BATF2 was upregulated during H1N1pdms infection. In previous analysis, BATF2 is an important regulator of the innate immune system and has high expression in human lung structural cells infected with influenza [67], indicating that BATF2 could play a critical role in host antiviral immune, but further studies are needed.

## 5. Conclusion

In our study, based on integration microarray datasets of the mouse lung infected with different H1N1pdms, host cells perform the similar immune response to different H1N1pdms. We further identified ten distinct DEGs (AREG, CXCL13, GATM, GPR171, IFI35, IFI47, IFIT3, ORM1, RETNLA, and UBD) differentially expressed genes during H1N1pdms infection compared with nonpandemic H1N1. These distinct dysregulated genes may have important regulation effects, and our future work will focus on revealing the function of these distinct dysregulated genes during influenza virus infection for the development of novel treatment strategies.

## Figures and Tables

**Table 1:** Microarray datasets of transcriptomic profiles.

Dataset	Platform	Influenza strain	Mouse	Time point (days)	Lethal infection
GSE43764	GPL13912	A/California/04/2009	6-week-old female BALB/c	3, 6 days [28]	No
GSE40091	GPL7202	A/California/04/2009	6- to 8-week-old female BALB/c	3, 5 days [29]	No
GSE63786	GPL7202	A/California/04/2009	5-week-old female C57BL/6Js	3, 5 days [30]	No
GSE67241	GPL6885	A/Jena/5258/09	7- to 8-week-old female BALB/c	3, 5 days [31]	No
GSE70882	GPL6246	A/California/07/2009	7-week-old female C57BL/6Js	3, 5 days [32]	No
GSE38112	GPL7202	A/BrevigMission/1/18	8- to 10-week-old female BALB/c	3, 5 days [33]	No
GSE70445	GPL7202	A/BrevigMission/1/18	8- to 9-week-old female BALB/c	3, 5 days [34]	No
GSE57008	GPL1261	A/Puerto Rico/8/34	5- to 7-week-old female C57Bl/6	3 days [35]	No
GSE99189	GPL16570	A/Puerto Rico/8/34	8- to 12-week-old female C57Bl/6	3 days [36]	No
GSE99190	GPL16570	A/Puerto Rico/8/34	8- to 12-week-old female C57Bl/6	3 days [36]	No
GSE77600	GPL11202	A/Puerto Rico/8/34	8- to 12-week-old female C57Bl/6	3 days [37]	No
GSE158270	GPL21163	A/Puerto Rico/8/34	6-week-old female BALB/c	5 days [38]	No
GSE62169	GPL16570	A/Puerto Rico/8/34	7- to 8-week-old female BALB/c	5 days [39]	No

## Data Availability

All data utilized in this manuscript are available online from their respective databases.

## Supplementary Materials

Supplementary Materials Supplementary data1: the DEG in A/California/04/2009 H1N1 infection. Supplementary data2: the DEG in two different strains of H1N1pdm09 (A/Jena/5258/09 and A/California/7/2009). Supplementary data3: the overlapped DEGs in all analysed datasets infected with H1N1pdm09 strains at 3dpi and 5dpi. Supplementary data4: H1N1pdm1918-induced DEGs. Supplementary data5: the DEGs induced by nH1N1pdm.

## References

[1] Elderfield R., Barclay W. (2011). Influenza pandemics. *Advances in Experimental Medicine & Biology*.

[2] Johnson N. P., Mueller J. (2002). Updating the accounts: global mortality of the 1918–1920 “Spanish” influenza pandemic. *Bulletin of the History of Medicine*.

[3] Taubenberger J. K., Morens D. M. (2010). Influenza: the once and future pandemic. *Public Health Reports*.

[4] Reid A. H., Fanning T. G., Hultin J. V., Taubenberger J. K. (1999). Origin and evolution of the 1918 “Spanish” influenza virus hemagglutinin gene. *Proceedings of the National Academy of Sciences*.

[5] Mena I., Nelson M. I., Quezada-Monroy F. (2016). Origins of the 2009 H1N1 influenza pandemic in swine in Mexico. *Elife*.

[6] Buchy P., Badur S. (2020). Who and when to vaccinate against influenza. *International Journal of Infectious Diseases*.

[7] Kumar S., Goicoechea S., Kumar S. (2020). Oseltamivir analogs with potent anti-influenza virus activity. *Drug Discovery Today*.

[8] Wang-Jairaj J., Miller I., Joshi A. (2022). Zanamivir aqueous solution in severe influenza: a global Compassionate Use Program. *Influenza and Other Respiratory Viruses*.

[9] Zhang C. X., Tu Y., Sun X. C. (2022). Peramivir, an anti-influenza virus drug, exhibits potential anti-cytokine storm effects. *Frontiers in Immunology*.

[10] Dufrasne F. (2021). Baloxavir marboxil: an original new drug against influenza. *Pharmaceuticals*.

[11] Lackenby A., Thompson C. I., Democratis J. (2008). The potential impact of neuraminidase inhibitor resistant influenza. *Current Opinion in Infectious Diseases*.

[12] Zhang M., Liu M., Bai S. (2021). Influenza A virus-host specificity: an ongoing cross-talk between viral and host factors. *Frontiers in Microbiology*.

[13] Staller E., Barclay W. S. (2021). Host cell factors that interact with influenza virus ribonucleoproteins. *Cold Spring Harb Perspect Med*.

[14] Mehrbod P., Ande S. R., Alizadeh J. (2019). The roles of apoptosis, autophagy and unfolded protein response in arbovirus, influenza virus, and HIV infections. *Virulence*.

[15] Martin-Sancho L., Tripathi S., Rodriguez-Frandsen A. (2021). Restriction factor compendium for influenza A virus reveals a mechanism for evasion of autophagy. *Nat Microbiol*.

[16] McKellar J., Rebendenne A., Wencker M., Moncorgé O., Goujon C. (2021). Mammalian and avian host cell influenza A restriction factors. *Viruses*.

[17] Killip M. J., Fodor E., Randall R. E. (2015). Influenza virus activation of the interferon system. *Virus Research*.

[18] Liu S. Y., Sanchez D. J., Aliyari R., Lu S., Cheng G. (2012). Systematic identification of type I and type II interferon-induced antiviral factors. *Proceedings of the National Academy of Sciences*.

[19] Desai T. M., Marin M., Chin C. R., Savidis G., Brass A. L., Melikyan G. B. (2014). Ifitm3 restricts influenza a virus entry by blocking the formation of fusion pores following virus-endosome hemifusion. *PLoS Pathogens*.

[20] Di-Pietro A., Kajaste-Rudnitski A., Oteiza A. (2013). TRIM22 inhibits influenza A virus infection by targeting the viral nucleoprotein for degradation. *Journal of Virology*.

[21] Herold S., Ludwig S., Pleschka S., Wolff T. (2012). Apoptosis signaling in influenza virus propagation, innate host defense, and lung injury. *Journal of Leukocyte Biology*.

[22] Liu X., Xu F., Ren L. (2021). MARCH8 inhibits influenza A virus infection by targeting viral M2 protein for ubiquitination-dependent degradation in lysosomes. *Nature Communications*.

[23] Skehel J. J., Wiley D. C. (2000). Receptor binding and membrane fusion in virus entry: the influenza hemagglutinin. *Annual Review of Biochemistry*.

[24] Lakadamyali M., Rust M. J., Zhuang X. (2004). Endocytosis of influenza viruses. *Microbes and Infection*.

[25] Zhao Y. X., Zou J. H., Gao Q. X., Xie S. S., Cao J. Y., Zhou H. B. (2021). CMAS and ST3GAL4 play an important role in the adsorption of influenza virus by affecting the synthesis of sialic acid receptors. *International Journal of Molecular Sciences*.

[26] Drews K., Calgi M. P., Harrison W. C. (2019). Glucosylceramidase maintains influenza virus infection by regulating endocytosis. *Journal of Virology*.

[27] Herold S., Mayer K., Lohmeyer J. (2011). Acute lung injury: how macrophages orchestrate resolution of inflammation and tissue repair. *Frontiers in Immunology*.

[28] Kiso M., Takano R., Sakabe S. (2013). Protective efficacy of orally administered, heat-killed Lactobacillus pentosus b240 against influenza A virus. *Scientific Reports*.

[29] Go J. T., Belisle S. E., Tchitchek N. (2012). 2009 pandemic H1N1 influenza virus elicits similar clinical course but differential host transcriptional response in mouse, macaque, and swine infection models. *BMC Genomics*.

[30] Shoemaker J. E., Fukuyama S., Eisfeld A. J. (2015). An ultrasensitive mechanism regulates influenza virus-induced inflammation. *PLoS Pathogens*.

[31] Manchanda H., Seidel N., Blaess M. F. (2016). Differential biphasic transcriptional host response associated with coevolution of hemagglutinin quasispecies of influenza A virus. *Frontiers in Microbiology*.

[32] Cole S. L., Dunning J., Kok W. L. (2017). M1-like monocytes are a major immunological determinant of severity in previously healthy adults with life-threatening influenza. *JCI Insight*.

[33] Jagger B. W., Wise H. M., Kash J. C. (2012). An overlapping protein-coding region in influenza A virus segment 3 modulates the host response. *Science*.

[34] Walters K. A., D’Agnillo F., Sheng Z. M. (2016). 1918 pandemic influenza virus and *Streptococcus pneumoniae* co‐infection results in activation of coagulation and widespread pulmonary thrombosis in mice and humans. *The Journal of Pathology*.

[35] Stegemann-Koniszewski S., Jeron A., Gereke M. (2016). Alveolar type II epithelial cells contribute to the anti-influenza A virus response in the lung by integrating pathogen- and microenvironment-derived signals. *mBio*.

[36] Suber F., Kobzik L. (2017). Childhood tolerance of severe influenza: a mortality analysis in mice. *American Journal of Physiology- Lung Cellular and Molecular Physiology*.

[37] Hatesuer B., Hoang H. T. T., Riese P. (2017). Deletion of and Genes in Mice Results in Altered Interferon Pathway Activation and Granulocyte-Dominated Inflammatory Responses to Influenza A Infection. *Journal of Innate Immunity*.

[38] Nishi A., Kaifuchi N., Shimobori C. (2021). Effects of maoto (ma-huang-tang) on host lipid mediator and transcriptome signature in influenza virus infection. *Scientific Reports*.

[39] Hrincius E. R., Liedmann S., Finkelstein D. (2015). Nonstructural protein 1 (NS1)-mediated inhibition of c-Abl results in acute lung injury and priming for bacterial co-infections: insights into 1918 H1N1 pandemic?. *The Journal of Infectious Diseases*.

[40] Ritchie M. E., Phipson B., Wu D. (2015). Limma powers differential expression analyses for RNA-sequencing and microarray studies. *Nucleic Acids Research*.

[41] Yu G., Wang L. G., Han Y., He Q. Y. (2012). clusterProfiler: an R package for comparing biological themes among gene clusters. *OMICS: A Journal of Integrative Biology*.

[42] Kanehisa M., Goto S. (2000). KEGG: Kyoto encyclopedia of genes and genomes. *Nucleic Acids Research*.

[43] Jassal B., Matthews L., Viteri G. (2020). The reactome pathway knowledgebase. *Nucleic Acids Research*.

[44] Shannon P., Markiel A., Ozier O. (2003). Cytoscape: a software environment for integrated models of biomolecular interaction networks. *Genome Research*.

[45] Herold S., Becker C., Ridge K. M., Budinger G. S. (2015). Influenza virus-induced lung injury: pathogenesis and implications for treatment. *European Respiratory Journal*.

[46] Martins R., Carlos A. R., Braza F. (2019). Disease tolerance as an inherent component of immunity. *Annual Review of Immunology*.

[47] Gounder A. P., Yokoyama C. C., Jarjour N. N., Bricker T. L., Edelson B. T., Boon A. C. M. (2018). Interferon induced protein 35 exacerbates H5N1 influenza disease through the expression of IL-12p40 homodimer. *PLoS Pathogens*.

[48] Yu Y., Xu N., Cheng Q. (2021). IFP35 as a promising biomarker and therapeutic target for the syndromes induced by SARS-CoV-2 or influenza virus. *Cell Reports*.

[49] Enomoto Y., Orihara K., Takamasu T. (2009). Tissue remodeling induced by hypersecreted epidermal growth factor and amphiregulin in the airway after an acute asthma attack. *Journal of Allergy and Clinical Immunology*.

[50] Bles N., Di Pietrantonio L., Boeynaems J. M., Communi D. (2010). ATP confers tumorigenic properties to dendritic cells by inducing amphiregulin secretion. *Blood*.

[51] Qi Y., Operario D. J., Georas S. N., Mosmann T. R. (2012). The acute environment, rather than T cell subset pre-commitment, regulates expression of the human T cell cytokine amphiregulin. *PLoS One*.

[52] Vermillion M. S., Ursin R. L., Kuok D. I. T. (2018). Production of amphiregulin and recovery from influenza is greater in males than females. *Biology of Sex Differences*.

[53] Monticelli L. A., Sonnenberg G. F., Abt M. C. (2011). Innate lymphoid cells promote lung-tissue homeostasis after infection with influenza virus. *Nature Immunology*.

[54] Eierhoff T., Hrincius E. R., Rescher U., Ludwig S., Ehrhardt C. (2010). The epidermal growth factor receptor (EGFR) promotes uptake of influenza A viruses (IAV) into host cells. *PLoS Pathogens*.

[55] Kazanietz M. G., Durando M., Cooke M. (2019). CXCL13 and its receptor CXCR5 in cancer: inflammation, immune response, and beyond. *Frontiers in Endocrinology*.

[56] Zeng Y. L., Lin Y. Q., Zhang N. N. (2016). CXCL13 chemokine as a promising biomarker to diagnose neurosyphilis in HIV-negative patients. *SpringerPlus*.

[57] Mehraj V., Ramendra R., Isnard S. (2019). CXCL13 as a biomarker of immune activation during early and chronic HIV infection. *Frontiers in Immunology*.

[58] Perreau M., Suffiotti M., Marques-Vidal P. (2021). The cytokines HGF and CXCL13 predict the severity and the mortality in COVID-19 patients. *Nature Communications*.

[59] Vuga L. J., Tedrow J. R., Pandit K. V. (2014). C-X-C motif chemokine 13 (CXCL13) is a prognostic biomarker of idiopathic pulmonary fibrosis. *American Journal of Respiratory and Critical Care Medicine*.

[60] Nakajima N., Sato Y., Katano H. (2012). Histopathological and immunohistochemical findings of 20 autopsy cases with 2009 H1N1 virus infection. *Modern Pathology*.

[61] Gerstmayer B., Küsters D., Gebel S. (2003). Identification of RELM*γ*, a novel resistin-like molecule with a distinct expression pattern☆. *Genomics*.

[62] Raes G., Noël W., Beschin A., Brys L., de Baetselier P., Hassanzadeh G. G. (2002). FIZZ1 and Ym as tools to discriminate between differentially activated macrophages. *Developmental Immunology*.

[63] Fan C., Meuchel L. W., Su Q. (2015). Resistin-like molecule *α* in allergen-induced pulmonary vascular remodeling. *American Journal of Respiratory Cell and Molecular Biology*.

[64] Ampomah P. B., Lim L. H. K. (2020). Influenza A virus-induced apoptosis and virus propagation. *Apoptosis*.

[65] Lee M. R., Shim D., Yoon J. (2014). Retnla overexpression attenuates allergic inflammation of the airway. *PLoS One*.

[66] Yu L., Wang L., Hu G. (2022). Reprogramming alternative macrophage polarization by GATM-mediated endogenous creatine synthesis: a potential target for HDM-induced asthma treatment. *Frontiers in Immunology*.

[67] Zhou A., Dong X., Liu M. Y., Tang B. (2021). Comprehensive transcriptomic analysis identifies novel antiviral factors against influenza A virus infection. *Frontiers in Immunology*.

